# Necrosis of the Alveolar Process: How Shall It Be Treated?

**Published:** 1894-11

**Authors:** J. Henry Morgan

**Affiliations:** Salem, Va.


ARTICLE VI.

### NECROSIS OF THE ALVEOLAR PROCESS: HOW SHALL IT BE TREATED?

BY J. HENRY MORGAN, D. D. S, SALEM, VA.

Necrosis is death of a bone or any portion of it; then whenever a part of the alveoli is, from any cause, deprived of vitality, it very soon becomes a source of irritation to the living parts to which it is attached, and the economy at once makes an effort to remove it; the dead portion of bone is separated from the living and is thrown off by exfoliation. The alveolar process is supplied with blood vessels and nerves like the other bones, yet their recuperative power is weak, and when a portion is destroyed by necrosis and exfoliated, the injury is not repaired by the process of nature, as it frequently is in the other bones. The cause of necrosis of the alveoli is sometimes obscure, seeming, in some cases, to be dependent on systemic influence, though it can often be clearly traced to dental irritation, followed by inflammation and death of the periosteum, and it frequently results from an immoderate or protracted use of mercurial medicine, and may be induced by a chronic ulcerated condition of the gums.

There is also a character of necrosis attacking the alveoli that sometimes occurs as a sequence of the exanthemata, such as small pox, measles or scarlet fever. It seems to occur mostly about the fifth or sixth year, just at a time when the jaw contains the whole of the temporary and the germs of the permanent set, more or less advanced. This affection presents itself within a few weeks after the disease has subsided—the gums becoming inflamed, swollen, tender, and separated from the teeth and alveoli, accompanied with a characteristic fetor of the breath. It appears remarkably symmetrical, occurring almost at the same time on both sides of the jaw, denuding the bone and leading to necrosis and the exfoliation of a considerable portion of it. This usually includes the whole depth of the process and developing teeth.

The tendency to necrosis exists at all periods of life, but especially in early youth, when it is most destructive and less amenable to treatment. The origin of this disease is not yet clearly shown. The usual explanation that it occurs as a consequence of any cause that sufficiently impedes the circulation, is true; but it is not so easy to assign a reason leading to the inference that its origin is idiopathic. Inflammatory conditions do not always produce it, or there would be little hope of ever saving a tooth that had an abscess. Unquestionably systemic conditions favor it, and it will begin from infection, though some question this. There is no doubt that when necrosis is once established in a bone it will pursue its course regardless of any kind of treatment or surgical interference. It is checked by natural processes, though just in what manner has not been explained in a satisfactory way. I believe the accepted opinion is that the periosteum or medullary membrane, as it may be, separates from the living bone, and, becoming inflamed, a quantity of ossific deposit is thrown out, which is converted into new bone, forming a covering for the dead portion, by which it is enclosed or invaginated. Some advance the idea that eventually pathogenic germs will be found to be the cause oi its origin. Should this be true, then its progress can be understood, and the reason shown why it is impossible to specify any period in its progress at which a sequestrum may be found separated from the living bone. One case that came under my observation commenced in the alveolus of an upper second molar; it continued slowly forwards involving all of the process to the central incisor, where it stopped. Just why it should end here remains a mystery.

The conditions that usually give rise to necrosis of the alveoli, are teeth, or roots of teeth with alveolar abscess, improperly filled pulp canals, or roots that have been improperly crowned, violence done in extracting, ulcerated conditions of syphilitic origin; then systemic conditions may be such as to favor the development of necrosis. I had charge of one case that was clearly the result of irritation due to an impacted lower wisdom tooth. The danger of extensive necrosis from abscessed teeth depends chiefly on two factors—the violence and duration of the inflammation and the heighth of temperature and upon the physical condition of the patient.

The character of infection as a cause of necrosis of the alveoli is important, but we have not enough knowledge of this variety upon which to rest an opinion. The preceding inflammation is attributable to infection from the root canal, either not filled or improperly so, *as with cotton*, or from the blood in a chronic or imperfectly heated abscess; however, in either case, the root canal or the tissue surrounding the apex of the root contains the exciting cause of the affection.

Now, in regard to the condition of the patient, in the majority of instances of necrosis from an alveolar abscess, or other cause, when it is extensive there is usually some constitutional taint, possibly temporary, and because of some systemic infection that would soon pass away under treatment, but which now favors the process of suppuration. These conditions are not often under the control of the practitioner, and an opinion as to their influence is not always possible. In these instances we often see necrosis occurring in different parts of the body, apparently without a local cause. When such a condition is recognized, it is bad practice to permit the inflammation to progress from an irritant that can be removed. A progressive necrosis always has a systemic cause.

In the treatment of these cases, so far as the portion of dead bone is concerned, the generally accepted opinion is that we shall wait for it to become separated from the living portion, for in attempting to remove it before then, there is always danger of destroying the new bone tissue being formed at the line of demarkation. When there is swelling and pus, the soft parts should be freely opened to give good drainage, and cleansed, as in this way the natural process is assisted. In a necrotic condition of the alveoli, after making an incision in the gum to release the pus, antiseptic and soothing mouth-washes should be prescribed. These will be found useful.

| | | | |
|---|---|---|---|
| R. | Acidi Boracici, | - - | dr. i. |
| | Glycerine, | - - | fl. ozs. ii. |
| | Zymocide, | - - - | fl. ozs. iii. |

M. Sig. One teaspoonful in a wineglass of water as often as may be indicated; or, where the gum is very tender, use

| | | | |
|---|---|---|---|
| R | Chloroform, | - - | fl. dr. ii. |
| | Tincture Arnicæ, | - - | fl. dr. ii. |
| | Listerine, | - - | fl. ozs. iii. |

M. Sig. Use as mouth-wash, diluted one-half, with warm water.

As a means to assist the separation of the necrosed portion aromaic sulphuric acid is recommended. It causes a rapid disintegration of the dead bone, so that it can be washed away. The rule in all cases of necrosis is to wait until the dead has been separated from the living bone. If it is torn away too soon necrosis will occur in the remaining portion.

There is a difference of opinion among practitioners of equally good authority, as to whether in necrosis of the alveoli the teeth or roots should be extracted, or wait for the separation of the sequestrum. Experience and observation seem to demonstrate that after a necrotic condition has once been established either by inflammation of the periosteum or ostitis, the teeth, *per se*, have no effect on the progress of the disease, and are not to be considered in the treatment. Now, in my judgment, as the teeth can do *no good* by being allowed to remain, and unquestionably act as an irritant to the necrosen portion, they should be extracted, not wfth the idea of averting the disease, but simply to remove all that can possibly be an irritant, taking the precautions that a surgeon does in any operation on necrosed tissue. The use of an antiseptic before, during and after the operation should not be omitted. The question of retaining or extracting the teeth should be well considered, as very much depends upon the condition of the tooth or teeth, and whether the disease is confined to the alveoli or has involved the body of the bone.

Now, just at this point, care is essential in forming a diagnosis, as the first is usually not very extensive and seems to be self-limiting, while the other may include the whole maxilla. When the alveolar process is only implicated the teeth can be retained, though it is not always advisable, as after having lost their attachment, they necessarily become a source of irritation. If a tooth is to be extracted, it can make no difference whether it is done during the inflammatory stage preceding suppuration or later, however, when extraction has been delayed until the suppurative stage is in full progress, and pus is being thrown out, the necessity for immediate extraction is not so urgent, and the operation may be postponed.

Though extraction should be the general rule, yet it should not be expected to avert an injury that has already been done. Then, until the dead bone becomes separated conservative treatment consists in seeing that all pus has a free outlet through the gums, and keep them open, and keep the mouth clean and aseptic by frequent use of such washes as

| | | |
|---|---|---|
| R | Zymocide, - - | fl. oz. i. |
| | Eucalyptol, - - | fl. drs. ii. |
| | Glycerine, - - - | fl. oz. i. |
| | Alcohol Dil, - - | fl. ozs. iii. |

M. Sig. Use as may be necessary.

| | | |
|---|---|---|
| R | Extract Hydrastic Canadence, | fl. oz. i. |
| | Listerine, - - - | fl. oz. i. |
| | Aquæ Rosæ, - - | fl. ozs. iv. |
| M. | Sig. Use as indicated. | |
| R | Extract Hamamelis Virg Dist., | fl. ozs. ii. |
| | Acidi Boracici, - - | dr. ss. |
| | Tincture Capsici, - | dr. ii. |
| | Menthol, - - - | grs. xx. |
| | Listerine, - - | fl. ozs. iii. |
| M. | Sig. Use as may be necessary. | |

Where the necrosis is extensive, the general health should receive attention, and good diet and tonics are recommended, such as the Cod Liver Oil preparations, and iron, quinine and strychnia.—*Southern Dental Journal.*

